# Transient Cortical Blindness in a Toddler With Heterozygous Ornithine Transcarbamylase Deficiency

**DOI:** 10.7759/cureus.20046

**Published:** 2021-11-30

**Authors:** Peter N Eskander, Sara S Romani

**Affiliations:** 1 Endocrinology and Metabolism, University of Missouri-Columbia School of Medicine, Columbia, USA; 2 Family Medicine, Kaiser Permanente Fontana Medical Center, Fontana, USA

**Keywords:** vision loss, metabolism, heterozygous, toddler, transient, urea cycle, glutamate, ornithine, cortical blindness, ornithine transcarbamylase deficiency

## Abstract

Ornithine transcarbamylase (OTC) deficiency is an incredibly rare disease in the subgroup of urea cycle disorders. Although typically seen in the neonate shortly after initiating high protein feeds (human breastmilk or infant formula), patients with partial/heterozygous deficiencies can often be diagnosed later in life with unique sequelae. One such manifestation is sudden, transient vision loss shortly after an initial episode of hyperammonemia in a patient without a known diagnosis of OTC deficiency. Only two such occurrences are documented in academic literature and both share many similar presenting features hinting that a hidden, but the consistent pathophysiologic mechanism of this disease is at play. Scarce research is available to propose a concise explanation; however, recent advancements in the literature point toward the brain’s inability to uptake glucose and convert it into glutamate in patients with partial OTC deficiency as a likely explanation.

## Introduction

Ornithine transcarbamylase (OTC) deficiency is one of several urea cycle disorders [1]. Urea cycle disorders are relatively rare, with a prevalence of 1/8,200 to 1/35,000; OTC deficiency is estimated to have a prevalence of 1/14,000 - 1/77,000 and is the most common urea cycle disorder [2]. In a healthy liver, the urea cycle converts nitrogen from peripherally catabolized proteins into water-soluble urea, which allows for seamless renal excretion of nitrogen. Deficiencies in any stage of the urea cycle will cause accumulation of substrates upstream of the faulty enzyme and ultimately results in an overabundance of the neurotoxin ammonia. The pathophysiologic basis of ammonia’s neurotoxicity is still the subject of great debate; however, some well-researched explanations have emerged. The typically observed consequences of encephalopathy in hyperammonemic patients are believed to largely be caused by ammonia’s ability to alter osmolarity and neuronal communication in the central nervous system. After crossing the blood-brain barrier, ammonia is taken up by astrocytes for detoxification where it is used as a substrate for the production of glutamine via glutamine synthetase. Glutamine is an osmolyte that increases the osmolarity of both the cerebrospinal fluid and astrocytes. This increase in osmolarity results in fluid shifts that result in cerebral edema and astrocyte swelling with subsequent dysfunction. Glutamine may also be taken up by neurons where it is converted to glutamate via glutaminase and released postsynaptically to later form neuroinhibitory molecules like gamma-aminobutyric acid (GABA).

OTC deficiency, similar to other urea cycle disorders, typically presents with symptoms of hyperammonemia after the first 24-48 hours following birth once the newborn has initiated high-protein feeds, especially in those with homozygous mutations [1]. Less common triggers for episodes of hyperammonemia include infection and use of certain medications, most notably valproic acid [3]. Initially, these symptoms are nonspecific - poor feeding, low core temperature, somnolence. These symptoms may then progress to lethargy, vomiting, seizures, and coma [1]. Hyperammonemia-induced cerebral edema may compress the brainstem and lead to deleterious effects on the patient’s respiratory drive and result in ventilatory alterations [1]. As cerebral edema worsens and pressure increases on the brain stem, patients will display abnormal posturing and appear encephalopathic. This progresses to hypoventilation, respiratory arrest, and severe neurologic disability if care is delayed [1]. Unlike other urea cycle disorders, OTC deficiency inheritance is considered to be X-linked dominant [2]. Therefore, it has been observed that symptoms tend to be more severe in males and variable in females. As such, the most severe cases of OTC deficiency typically manifest in neonatal males, while afflicted females can present later in life.

Although neurologic abnormalities are a common manifestation of OTC deficiency, vision loss is a particularly atypical symptom that is exceptionally rare [3]. An in-depth literature review spanning over the last 50 years yielded only three other case reports in the academic literature [3-5]. Two cases were in young females who suffered their vision loss 1-2 weeks after their initial diagnosis of OTC deficiency was made when they were incidentally found to be hyperammonemic [3,4]. The third case was in an adult male who developed his vision loss during an episode of hyperammonemia and neuropsychiatric manifestations, which differs significantly from this case in terms of presentation [5]. This interesting case aims to describe the presentation, findings, and treatment of a patient with OTC deficiency associated with sudden vision loss, as well as proposes a possible pathophysiologic mechanism for this phenomenon.

## Case presentation

Initial OTC deficiency diagnosis

A three-year-old female with a history of mild receptive and expressive language delay initially presented to the emergency department with three days of altered mental status. She was found to have elevated ammonia on admission (207 mmol/L; normal <34) and a plasma amino acid panel positive for glutamine, alanine, and methionine elevation and decreased citrulline, arginine, and ornithine. Urine orotic acid was also elevated, which supported a running diagnosis of OTC deficiency. Cranial imaging at that time, including magnetic resonance imaging (MRI) brain and brainstem, was unremarkable. She was discharged on sodium benzoate and a low protein diet. Shortly after her discharge, gene sequencing revealed a heterozygous variant c.205C>T p.Gln69* in the OTC gene, a pathogenic mutation most consistent with OTC deficiency. Her medication regimen was changed to daily glycerol phenylbutyrate (Ravicti) and Citrulline supplementation with ammonia levels subsequently normalizing.

Presentation of vision loss

One week after her initial OTC deficiency diagnosis, the patient’s father expressed concerns about the patient’s vision, stating she was walking with her hands outstretched and appears to not see items presented in front of her, like food and falling objects. For the next two days, her parents noted that the patient’s vision continued to decline. She exhibited unresponsiveness to light, inability to identify people or objects in front of her, and worsening instability when walking.

She was seen by a pediatric ophthalmologist who was unable to assess her visual acuity but found her to have no response with intense illumination with indirect ophthalmoscope light. Slit-lamp and fundus exam were similarly unremarkable but she had a severely depressed threat response. Extraocular movements were intact and both pupils were reactive to light, although the right reacted more sluggishly. Minimal anisocoria was observed (right > left). She was diagnosed with bilateral cortical vision loss and, after discussion with the metabolic department and pediatric neurology, was scheduled to have a repeat MRI brain and orbits with and without contrast for the evaluation of optic neuritis. Special attention was paid to the FLAIR sequencing and occipital lobes based on previous case reports of vision loss in patients with OTC deficiency.

Inpatient course

**Figure 1:**
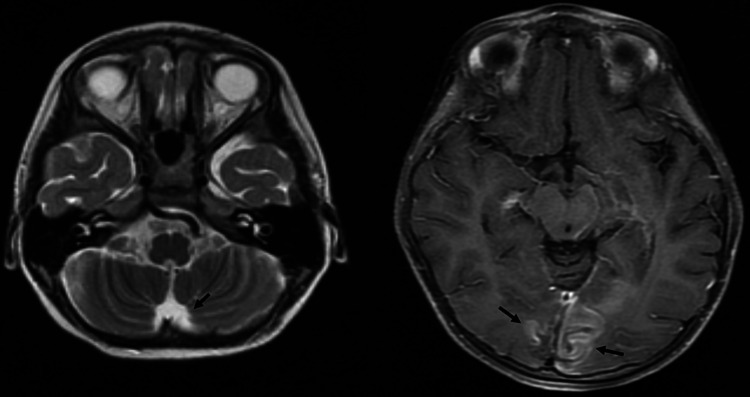
MRI of the brain at the level of the occipital lobes with T1-weighted imaging (left) and T2-weighted imaging (right), demonstrating nonspecific abnormal enhancement involving the bilateral occipital lobes without pronounced associated FLAIR abnormality.

**Figure 2:**
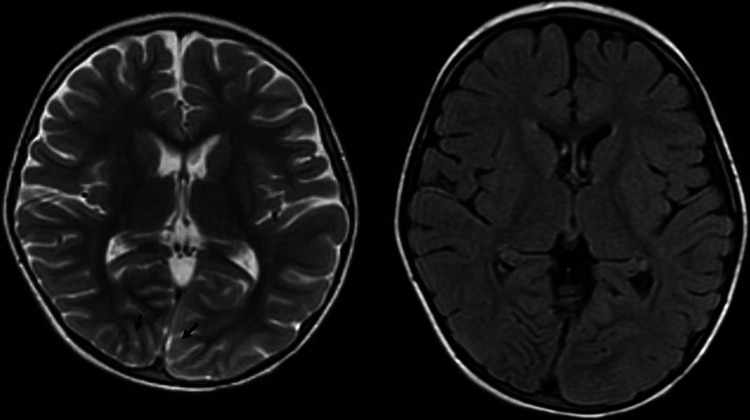
MRI imaging of the brain at the level of the cuneus gyrus demonstrating cortical enhancement of the lingual and cuneus gyri bilaterally with T2-weighted imaging (left). There was no associated restricted diffusion or FLAIR signal abnormality in this region (right).

The patient was directly admitted and noted to have normal vital signs and ammonia levels. Plasma arginine and citrulline levels were within normal limits, ornithine, glutamine, and alanine levels were slightly elevated, and valine levels were slightly low at the time of admission. MRI showed no abnormalities in the course, caliber, and signal intensity of the optic nerves. The remainder of the contents of the orbits including the lacrimal glands, vascular structures, and extraocular muscles were normal in appearance. MRI was positive for nonspecific abnormal enhancement involving the bilateral occipital ​lobes without pronounced associated FLAIR abnormality (Figure 1). MRI also demonstrated cortical enhancement involving the lingual and cuneus gyri bilaterally (Figure 2, left). There was no associated restricted diffusion or FLAIR signal abnormality in this region (Figure 2, right). However, there was mild prolongation involving the parietal gyri just superior to this region. There may have been minimal T2 prolongation involving the bilateral subcortical occipital white matter in the prior study. There was mild gyral restricted diffusion involving the left motor strip with corresponding mild T2 prolongation.

Following MRI and sedation, the patient had incidental onset of complex partial seizures involving twitching of her mouth and tonic/clonic movements of her right upper extremity. She received abortive medications and a loading dose of levetiracetam. Electroencephalogram (EEG) was positive for epileptiform discharges correlating with right mouth twitching and she was started on maintenance doses of levetiracetam and clonazepam with decreasing frequency and duration of episodes. She has since been seizure-free. The seizure activity was attributed to a sequela of anesthesia.

This episode of sudden vision loss was self-limited and required no other intervention. Neuroimaging six weeks following her hospitalization was normal. These findings are similar to two other noted cases of acute onset vision loss in females with OTC deficiency about 1-2 weeks after an initial episode of hyperammonemia without concurrent elevation in ammonia levels at the time of vision loss.

## Discussion

Neuropsychiatric manifestations of OTC deficiency, including developmental delay, intellectual disability, and attention deficit hyperactivity disorder, are well-established consequences of the illness. A far less common repercussion is that of transient vision loss.

In the setting of the amino acid derangements that accompany OTC deficiency, plasma arginine levels must be evaluated since low levels have been associated with visual disturbances in mitochondrial encephalomyopathy with lactic acidosis and stroke-like episodes (MELAS) [6]. This was promptly ruled out with normal arginine levels in this patient, but still, merits consideration since OTC deficiency is commonly associated with low arginine levels due to disruption of the urea cycle [7]. Another amino acid worth evaluating is ornithine. Hyperornithinemia, in the context of other illnesses like gyrate atrophy, has caused considerable damage to the retinas resulting in progressive, permanent blindness in adulthood [8]. The pathophysiologic basis of blindness in gyrate atrophy differs from that of OTC deficiency due to its reversibility, acute nature, and characteristic imaging, however, this patient did have mildly elevated ornithine levels at the time of her vision loss.

The two recorded case reports of this rare phenomenon documented incredibly similar imaging findings to those seen in this patient - ﻿ bilateral cortical enhancement of the posterior and medial occipital lobe, specifically along the lingual and cuneus gyri [3,4]. This is potentially explained by the cerebrovascular hyperreactivity resulting from hyperammonemia secondary to OTC deficiency, which may result in ischemic changes [9,10]. The increase in extracellular glutamate that results from the detoxification of ammonia, as described above, causes overstimulation of N-methyl-D-aspartate (NMDA) receptors which in turn causes overproduction of the highly vasoactive compound nitric oxide (NO). NO causes vasodilation which contributes to the ischemic changes in the brain parenchyma associated with OTC deficiency. However, this patient’s brain imaging and subsequent blindness uniquely occurred in the absence of hyperammonemia, similar to the two previously reported cases, which rules down the possibility that the imaging findings were caused by ischemic changes. Novel research studying the effect of partial OTC deficiency on the cerebral uptake of glucose and conversion to glutamate revealed that these patients have markedly decreased functionality [11]. Despite the small sample size, these findings may indicate a target for the treatment of OTC deficiency in patients with ophthalmologic manifestations despite adequate control of ammonia, as well as, another potential explanation for this rare occurrence. Prognostic factors associated with MRI abnormalities in the setting of urea cycle disorders have been studied [12]; however, these studies continue to explore the consequences of hyperammonemia as that is the most prominent feature of this disease. Our findings highlight the necessity for more literature on the effects of OTC deficiency on the brain with the goal that this case report will draw more attention to this orphan diagnosis in the future.

## Conclusions

Transient cortical blindness may occur in young females with heterozygous OTC deficiency weeks after an initial episode of hyperammonemia. After formally establishing the diagnosis of OTC deficiency with laboratory and genetic evaluation, the diagnosis of transient cortical blindness secondary to OTC deficiency was confirmed via the characteristic imaging findings in Figures 1, 2 that were shared amongst the only other two case reports in the academic literature. These findings include abnormal enhancement of the occipital ​lobes bilaterally and cortical enhancement of the lingual and cuneus gyri. It is most likely this patient’s vision improved with the continuation of her outpatient medical regimen, but it is still worth noting that she did receive anti-seizure medications during her hospital course. However, these medications are not known to have effects with regard to cortical blindness. Amino acid abnormalities such as low arginine, elevated ornithine, and impaired glutamate metabolism are reasonable future directions for studying the pathophysiology of transient visual disturbances in OTC deficiency. Although OTC deficiency-associated cortical blindness is rare, it is prudent to consider this condition in the differential diagnosis of a young female presenting with new-onset vision loss.
